# Primary Subcutaneous Ewing Sarcoma Presented as Pseudo Aneurysmal Subcutaneous Tumor

**DOI:** 10.4021/wjon2010.01.1205

**Published:** 2010-02-01

**Authors:** Faten Hammedi, Sonia Ziadi, Mounir Trimeche, Wafa Jomaa, Baddredine Sriha, Sadok Korbi

**Affiliations:** a Department of Pathology, Farhat Hached Hospital, Sousse, Tunisia

**Keywords:** Extraskeletal Ewing’s sarcoma, Subcutaneous tissue, Histopathology, Immunohistochemical

## Abstract

**Background:**

Extraskeletal Ewing's sarcoma is a rare malignant tumor of mesenchymal origin, which is histologically similar to primary osseous Ewing's sarcoma. It has been well described in deep soft tissues. However, location in cutaneous or subcutaneous tissue has rarely been reported. Being seen principally in children, it can be seen, rarely, in old men.

**Case report:**

We present a case of subcutaneous Ewing sarcoma within the left shoulder of a 49-year-old man, without osseous involvement. Physical examination suggested a vascular tumor. Histologically, it was a small round cell tumor that marked strongly for CD99. The diagnosis of subcutaneous Ewing sarcoma was performed.

**Conclusion:**

Ewing sarcoma is a rare malignant small round cell tumor of the skin and subcutaneous tissue. It should be differentiated from other cutaneous neoplasms composed of small round cells.

## Introduction

In 1921, James Ewing [1] first described the tumor of bone that is now known as Ewing’s sarcoma (ES). Although primary osseous ES was a common tumor, primary ES of skin and subcutaneous tissue is rare neoplasm. This tumor is histologically indistinguishable from ES of bone. That is why the osseous and extraosseous lesions comprise the Ewing’s sarcoma family tumors (ESFT) [2]. Primary cutaneous or subcutaneous ES is mainly seen in children and young adults (median age 18 years), but it occasionally afflicts elderly individuals. There is no significant sex predilection.

The superficial variant may be less aggressive than the more common bony and deep soft tissue counterparts with an apparently favorable outcome [3-5]. This tumor, being seen principally in children, afflicts, occasionally, elderly individuals. We present the case of a 49-year-old man, who presented with extraskeletal Ewing's sarcoma involving the skin, with discussion of the literature and problems concerning differential diagnosis of small cell malignancies in the skin.

## Case Report

**Figure 1:**
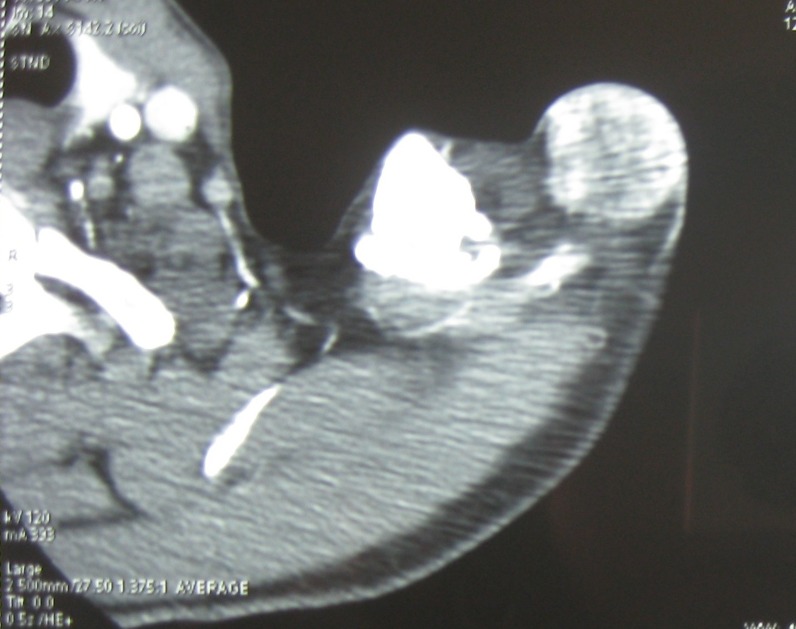
Angioscan: subcutaneous hypervascular and well circumscribed mass of the shoulder, without osseous involvement (arrow).

**Figure 2:**
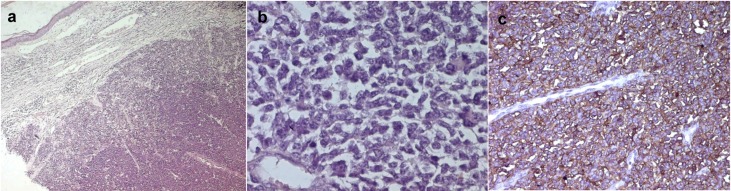
a: Hematoxylin and eosin staining showing dermal small cells proliferation with sharp circumscription (Magnification x 200); b: Hematoxylin and eosin staining showing a monotonous sheet of malignant small round to oval cells with nuclear atypia (Magnification x 200); c: Immunohistochemistry showing positivity of the tumor cells for CD99 (Magnification x 200).

A 49-year-old man presented with a six-month history of a dermal and subcutaneous tumor of the left shoulder, without osseous involvement. Physical examination suggested a vascular tumor. An angioscan found a subcutaneous hypervascular and well circumscribed mass of the shoulder, without osseous involvement (Fig. 1). The rest of the physical examination and imaging exploration were normal. A surgical excision of the mass was performed. Macroscopically, it was a well circumscribed hemorrhagic lesion, which measured 6 x 3 x 1.5 cm. Histopathological examination showed a small round cell malignant tumor with extensive geographic necrosis, located in the dermis (Fig. 2a). Cellular atypia was seen (Fig. 2b) and occasional mitoses were seen with a count of four mitoses per 10 high power fields. Thin walled capillary often accompanied tumor cells. Immunohistochemical studies showed that the tumor cells stained positive for CD99 (MIC2) (Fig. 2c). No positive immunostaining was seen for chromogranin and S-100. After surgery, the patient received chemotherapy.

## Discussion

Cutaneous or subcutaneous Ewing's sarcoma/malignant primitive neuroectodermal tumor (PNET) may rarely occur as a primary, superficially based neoplasm in children and young adults. About 82 cases of cutaneous or subcutaneous ES were reported in the literature. These tumors occur most frequently during the second decade of life (64%) [2]. The mean age at diagnosis was 15 years (22 months to 81 years). Our patient was a 49-year-old man. The tumors were observed throughout the body and were frequently seen in the sole of the foot. There were no specific symptoms. That is why confirmation of the diagnosis was made by histological examination and immunohistochemical examination. Not infrequently, they are clinically misdiagnosed as benign tumors, cysts or pseudo aneurysmal tumor [6].

In our case, physical examination and imaging exploration suggested a vascular tumor (an angioma). In fact, prominent fibrovascular septa are present in most lesions, like our case, and can mime a vascular tumor. The histological image of cutaneous or subcutaneous ES is a small blue round cell with a scanty cytoplasm, and it is often confused with other small round cell tumors. Prominent fibrovascular septa are present in most lesions, like our case. The cells are usually periodic acid-Schiff (PAS)-positive, indicating the presence of glycogen granules [2]. Characteristically, the tumor cells were positive for CD99 (MIC2 gene product), ß2 microglobulin, FLI-1 gene product and vimentin [3]. Cutaneous or subcutaneous ES should be differentiated from the other small round cell tumors of the skin such as lymphoblastic lymphoma, Merkel cell carcinoma and basaloid cell tumors, which are commonly seen in the skin (such as carcinomas of appendageal origin) and rarely from metastatic tumors in the skin (such as rhabdomyosarcoma and neuroblastoma). The confrontation of the pathological examination with immunohistochemistry may be helpful for the diagnosis. The negative of leukocyte common antigen, CD30, myosin, actin, myoglobulin, neurofilament, neuron-specific enolase and the S-100 protein can exclude lymphoblastic lymphoma, rhabdomyosarcoma and neuroblastoma [7, 8]. Basaloid adnexal tumors of the skin are negative for CD99 [9]. Our patient was diagnosed with extraskeletal Ewing's sarcoma based on the results of extensive immunohistochemical staining as described above. Cutaneous or subcutaneous ES shows a distinctive reciprocal chromosome translocation t (11; 22) (q24; q12) [2, 10]. Treatment for cutaneous Ewing's sarcoma, though not codified, consists of a large surgery associated with polychemotherapy and/or radiotherapy [2, 3, 9]. More cases compiled from the literature suggest that superficial variant of soft tissue ES has a more favorable prognosis than of other forms of ES [3-5], probably because they are detected early and can be resected adequately, in view of the easily accessible superficial location [9]. Other prognostic factors of a favorable outcome were reported in the literature such as young age at diagnosis, small tumor size, lack of metastasis, good response to treatment and low serum lactate dehydrogenase (LDH) levels [2].

In conclusion, though rare, primary cutaneous ES/PNET should be considered in the differential diagnosis of cutaneous ‘small blue cell tumors’. The number of cases of cutaneous ES/PNET studied so far is rather small, and no firm conclusion can be drawn about their behavior. Further cases are needed to more evaluate the prognosis of these tumors. However, the prognosis of primary cutaneous ES/PNET appears to be more favorable than extracutaneous ES/PNET.
